# Technospheric Mining of Rare Earth Elements from Bauxite Residue (Red Mud): Process Optimization, Kinetic Investigation, and Microwave Pretreatment

**DOI:** 10.1038/s41598-017-15457-8

**Published:** 2017-11-10

**Authors:** Sable Reid, Jason Tam, Mingfan Yang, Gisele Azimi

**Affiliations:** 1 Laboratory for Strategic Materials, Department of Chemical Engineering and Applied Chemistry, University of Toronto, Toronto, ON M5S 3E5 Canada; 2 Department of Materials Science and Engineering, University of Toronto, Toronto, ON M5S 3E4 Canada

## Abstract

Some rare earth elements (REEs) are classified under critical materials, i.e., essential in use and subject to supply risk, due to their increasing demand, monopolistic supply, and environmentally unsustainable and expensive mining practices. To tackle the REE supply challenge, new initiatives have been started focusing on their extraction from alternative secondary resources. This study puts the emphasis on technospheric mining of REEs from bauxite residue (red mud) produced by the aluminum industry. Characterization results showed the bauxite residue sample contains about 0.03 wt% REEs. Systematic leaching experiments showed that concentrated HNO_3_ is the most effective lixiviant. However, because of the process complexities, H_2_SO_4_ was selected as the lixiviant. To further enhance the leaching efficiency, a novel process based on microwave pretreatment was employed. Results indicated that microwave pretreatment creates cracks and pores in the particles, enabling the lixiviant to diffuse further into the particles, bringing more REEs into solution, yielding of 64.2% and 78.7% for Sc and Nd, respectively, which are higher than the maximum obtained when HNO_3_ was used. This novel process of “H_2_SO_4_ leaching-coupled with-microwave pretreatment” proves to be a promising technique that can help realize the technological potential of REE recovery from secondary resources, particularly bauxite residue.

## Introduction

Rare earth elements (REEs) have unique physicochemical properties that make them indispensable in many emerging critical and green innovations. The performance of REE in the high-tech products is irreplaceable by other materials; hence their demand is increasing steeply, and some are classified under critical materials^1,2^. REEs are not rare, but their supplies are insecure because of geologic scarcity, extraction difficulties, and dependence on sources in politically volatile countries^2–6^. According to the historic data, for some REEs, the supply will not sustain the increasing demand due to the time lags involved in bringing new production capacity online^2^, which on the positive side stimulated other countries to look for alternative “secondary resources” for REEs to tackle their supply challenge^7^.

Technospheric mining of REEs from secondary resources can incorporate direct recycling of end-of-life REEs containing products, such as permanent magnets^8^ and lamp phosphors^9^, landfill mining of historic urban solid waste^10^, and recovery from stocks of landfilled industrial process residues^5^, such as phosphogypsum, bauxite residue (red mud), mine tailings, and metallurgical slags that can secure an independent source of REEs for resource-poor countries to satisfy their REEs demand^5^.

Bauxite residue is the solid residue generated in the Bayer process for aluminum production. About 0.7–2 tons of bauxite residue is generated for each ton of alumina produced^11^, and the annual global production of bauxite residue is about 140 million tons^12^, while 2.7 × 10^9^ tons were stockpiled by 2011^13^. The cumulative amount of bauxite residue generated by 2015 is estimated to be close to 4 × 10^9^ tons^14^. It is known that bauxite ores contain REEs, as ions adsorbed on surface of minerals or as REE replacing similar ions in some minerals (isomorphous substitution)^15^. The REEs present in the bauxite ore end up in bauxite residue with an enrichment factor of two; because they are associated with iron and titanium minerals that remain unchanged in the Bayer process^5^. Among different REEs present in red mud, scandium is the most strategic one because of the game-changing properties it offers to the modern society.

With an average crustal abundance of around 22 ppm, scandium is not particularly rare. In fact, it is more abundant than lead, mercury, and precious metals^16^. Despite this fairly common occurrence, scandium rarely concentrates in nature, as it lacks affinity to combine with ore-forming anions. Hence, there has been no record of scandium deposits with concentrations over 100 ppm. Currently there is no dedicated scandium mine and it is mainly produced as a byproduct during the processing of various ores or recovered from previously processed tailings or residues. The global production rate of scandium was estimated to be about 15 tonnes per year^17^. Resources with scandium content between 20 and 50 ppm can be considered as an ore, which makes bauxite residue a suitable source for this element.

Despite the scarcity and high cost of scandium, there has been a significantly growing interest in the element. One of the main interests is the alloy of scandium into aluminum products, which results in stronger, weldable, more corrosion resistant, and heat tolerant aluminum products. Aircraft manufacturers are particularly interested in Al-Sc alloys because the ability to employ weldable structures could reduce aircraft weights by 15–20%. Considering the global annual market for aluminum, if only a small fraction (0.1%) absorbs scandium in alloys at a 0.5% level, it will result in 350 tonnes annual global demand for scandia (Sc_2_O_3_).

There have been many studies on the extraction of REEs, and particularly scandium, from bauxite residue^5,16^. According to the literature, the leaching process depends on the type of bauxite residue, because mineralogical composition and morphological distribution of bauxite residue determine the best leaching agent. Hence, the REE recovery process from bauxite residue must be tailored for a given source^5^. Most of the previous studies were focused on Greek, Jamaican, Indian, Australian, Hungarian, Russian, and Chinese bauxite residue. Nevertheless, no extensive study was published on Canadian bauxite residue^16^. In a systematic study, the effect of HCl, H_2_SO_4_, and HNO_3_ on the leaching efficiency of REEs from Greek bauxite residue has been investigated and has shown that dilute HNO_3_ (0.5 M) is the best leachant at 25 °C with a solid to liquid ratio of 1/50^18^. This process has been tested at a pilot scale and optimized^19^. The main disadvantage of HNO_3_ leachant is the difficulty of removing the nitrates ions from the remaining residue. If the residue is flushed with excess water, a large volume of wastewater with high concentration of nitrate ions will be generated. Since most nitrate salts are soluble, removing these ions from the wastewater through precipitation is challenging. Furthermore, nitric acid is very corrosive, which results in increased capital and operating costs for the process. In another study performed on Australian bauxite residue, it has been shown that dilute (0.5 M) H_2_SO_4_ with a solid to liquid ratio of 1/20 is the most efficient case; however, the maximum scandium leaching efficiency achieved was 47.6%^14^. There has also been a process developed by Orbite Technology Inc., in which bauxite residue is leached with a concentrated HCl (18–33 wt%) solution in an autoclave at a temperature of 140–170 °C^20^. In this process, all components (except titanium and silicon dioxide) are dissolved. Aluminum, iron, and magnesium are recovered by treating the solution with HCl gas and subsequent removal steps. The REEs are separated from the leach solution by solvent extraction. Although promising, there are some drawbacks, mainly due to handling of highly corrosive HCl, which requires glass-lined reactors as well as valves and pipes made of high-performance chemically resistant polymers resulting in increased capital, operating, and maintenance costs. Furthermore, the residue of this process is very acidic (considering the very high concentration of HCl used) and its handling and storage could be challenging.

In this study, we developed an innovative process to recover REEs from Canadian bauxite residue by enhancing the leaching efficiency with a microwave pretreatment. To systematically develop the REE recovery process from bauxite residue, three different leaching agents (H_2_SO_4_, HCl, and HNO_3_) under various operating conditions in terms of temperature, concentration, solid to liquid ratio (S/L), and residence time with and without microwave pretreatment were investigated and optimal operating conditions were determined. We expect the findings of this study help realize the recovery of REEs from bauxite residue and address sustainability challenges in the REE industries.

## Results and Discussion

### Characterization of bauxite residue

**Figure 1:**
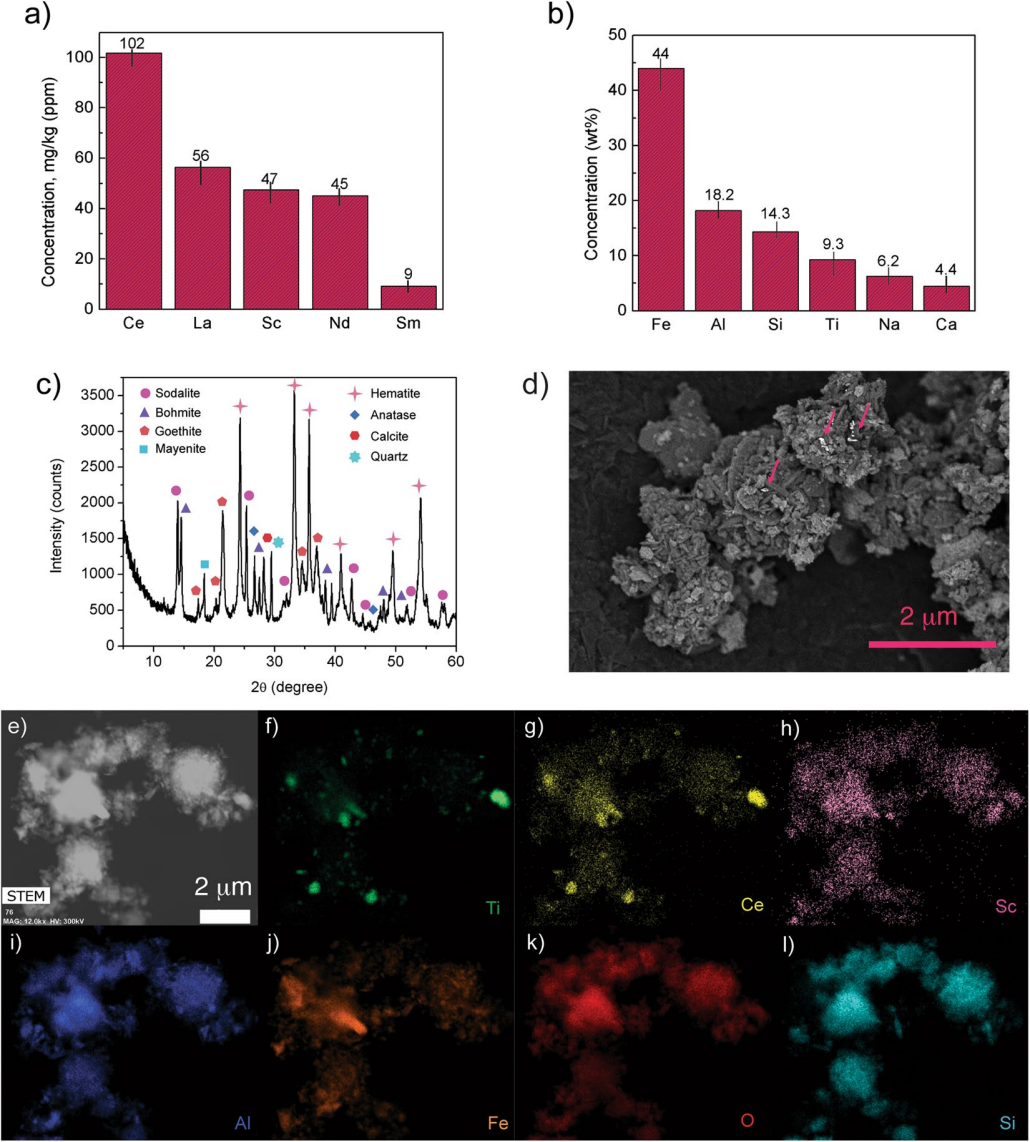
Bauxite residue characterization results. (**a**) Aqua regia digestion - ICP-OES results indicating REEs composition. (**b**) XRF elemental composition. Error bars represent the standard error of the mean for ten replicates (**c**) X-ray diffractogram. (**d**) BSE image of the bauxite residue particle; bright spots are the high atomic number REEs. (**e**–**l**) STEM image and EDS elemental mapping of the bauxite residue particle.

The chemical composition of bauxite residue was analyzed with X-ray fluorescence (XRF), as well as with aqua regia digestion and inductively coupled plasma optical emission spectroscopy (ICP-OES). The main REEs present are cerium (Ce), lanthanum (La), neodymium (Nd), scandium (Sc), and samarium (Sm) with a total concentration of about 0.03 wt% (Fig. 1a). The main impurities are iron (Fe), aluminum (Al), silicon (Si), titanium (Ti), sodium (Na), and calcium (Ca) (Fig. 1b). The crystal structure of bauxite residue was characterized by X-ray diffraction (XRD) and it was found that hematite (Fe_2_O_3_, 33 wt%), goethite (FeO(OH), 23 wt%), sodalite (Na_8_Al_6_Si_6_O_24_, 15 wt%), boehmite (AlO(OH), 10%), anatase (TiO_2_, 5.7 wt%), quartz (SiO_2_, 5 wt%), mayenite (Ca_12_Al_14_O_32_, 4 wt%) and calcite (CaCO_3_, 3 wt%) are the main phases (Fig. 1c). The surface morphology of bauxite residue particles was characterized by scanning electron microscopy (SEM) and REEs were detected as bright spots (Fig. 1d) in the atomic number contrast backscattered electron (BSE) image. The bauxite residue particles were also characterized by scanning transmission electron microscopy (STEM) equipped with energy dispersive X-ray spectroscopy (EDS) and elemental mapping was obtained (Fig. 1e–l). The result is in agreement with the mineralogy of bauxite residue^5^; Ce was found to be associated with titanium, whereas scandium was found to be amalgamated with iron and aluminum compounds.

### Acid selection for REEs leaching from bauxite residue

**Figure 2:**
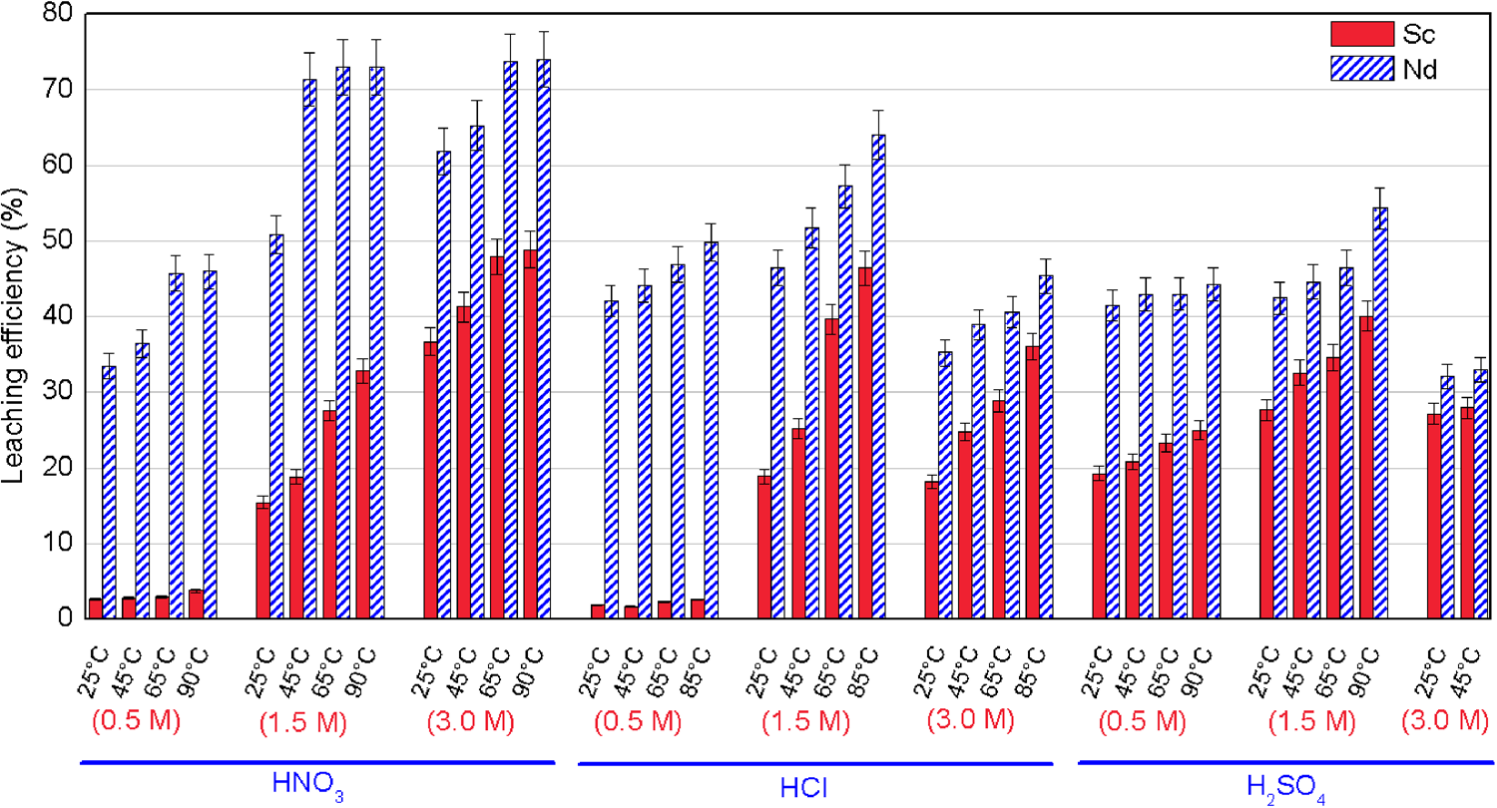
Leaching efficiency of neodymium (Nd) and scandium (Sc) using HNO_3_, HCl, and H_2_SO_4_ at different temperatures (25–90 °C) and concentrations (0.5, 1.5, 3.0 M) within 30 min residence time. Error bars represent the standard error of the mean for four replicates.

To choose the best leaching agent and optimum operating conditions, a systematic investigation was performed using HNO_3_, HCl, and H_2_SO_4_ at 0.5, 1.5, and 3 M and four different temperatures (25, 45, 65, and 90 °C) at a S/L of 1/15. Based on the REEs prices released by minerals prices, scandium accounts for about 99% of the overall value of REEs in bauxite residue, and the next critical element is neodymium. Thus, the emphasis was focused on these two elements. Figure 2 presents the leaching efficiency of neodymium and scandium for the three acids at various concentrations and temperatures. In all cases, the leaching efficiency increases with increasing temperature. When HNO_3_ was used as the leaching agent, the leaching efficiency increased with increasing acid concentration, reaching 49% and 79% for Sc and Nd, respectively. In the cases of HCl, the leaching efficiency increased with increasing acid concentration from 0.5 M to 1.5 M, but decreased at 3 M. The solubility decrease in concentrated HCl solutions can be explained by the concept of solvation: as the concentration of electrolyte increases, fewer water molecules can participate in the dissolution process because they are tightly held (solvated) by cations and anions in the solution, this is known as the salting-out effect^21^. The maximum leaching efficiency of Sc and Nd were 46% and 64%, respectively. Similar to the HCl case, the leaching efficiency of Sc and Nd in H_2_SO_4_ increased from 0.5 M to 1.5 M and then decreased at 3 M acid concentration, again because of salting out effect. The maximum leaching efficiency for Sc and Nd was 40% and 54%, respectively. These results indicate that highest leaching efficiency was obtained with 3 M HNO_3_ at 90 °C, followed by 1.5 M HCl and then 1.5 M H_2_SO_4_. However, beside higher price, there are challenges involved with using HNO_3_ as the leaching agent, including the difficulty of removing nitrate ions and handling of process residue as well as high corrosivity. Using HCl as the leaching agent also has some disadvantages, including higher reactant price as well as higher capital, operating, and maintenance costs and process complexities due to high corrosivity challenges of HCl. Furthermore, comparing the leaching efficiencies for the HCl and H_2_SO_4_ cases indicates that there is only 6% and 10% increase in the leaching efficiency of Sc and Nd, respectively. Therefore, it is not worth choosing HCl as the leaching agent considering the aforementioned disadvantages. In addition to Nd and Sc, we looked at the leaching efficiency of impurities, i.e., Al, Fe, and Ti, since they need to be removed downstream. The leaching efficiencies for these elements were 50%, 9.6%, and 8.4% for 3 M HNO_3_; 53%, 4.3%, and 8% for 1.5 HCl, and 50%, 3.3%, and 6.7% for 1.5 M H_2_SO_4_, respectively. Therefore, among the cases studied, 1.5 M H_2_SO_4_ at 90 °C was selected and further investigations were performed to improve the leaching efficiency for this case.

### Optimum solid to liquid ratio

**Figure 3:**
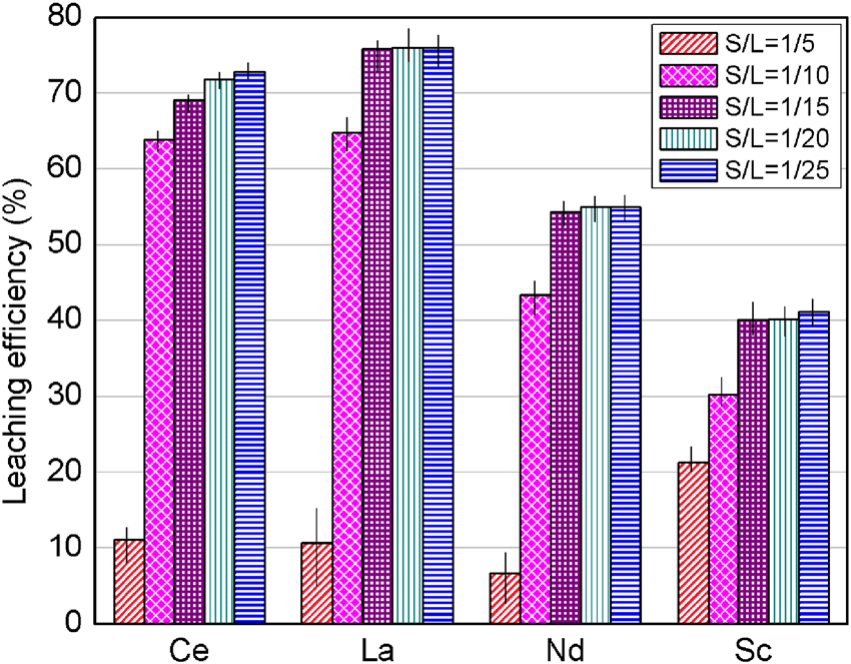
Effect of solid to liquid ratio (S/L) on the leaching efficiency of cerium (Ce), lanthanum (La), neodymium (Nd), and scandium (Sc) using 1.5 M H_2_SO_4_ at 90 °C within 30 min residence time. Error bars represent the standard error of the mean for four replicates.

Figure 3 presents the leaching efficiency of cerium, lanthanum, neodymium, and scandium for 1.5 M H_2_SO_4_ at 90 °C. As demonstrated in Fig. 3, the leaching efficiency increases by decreasing S/L from 1/5 to 1/15 and then remains unchanged beyond that. Hence, it can be concluded that S/L of 1/15 is the optimum case for maximizing the leaching efficiency in this system.

### Microwave pretreatment

**Figure 4:**
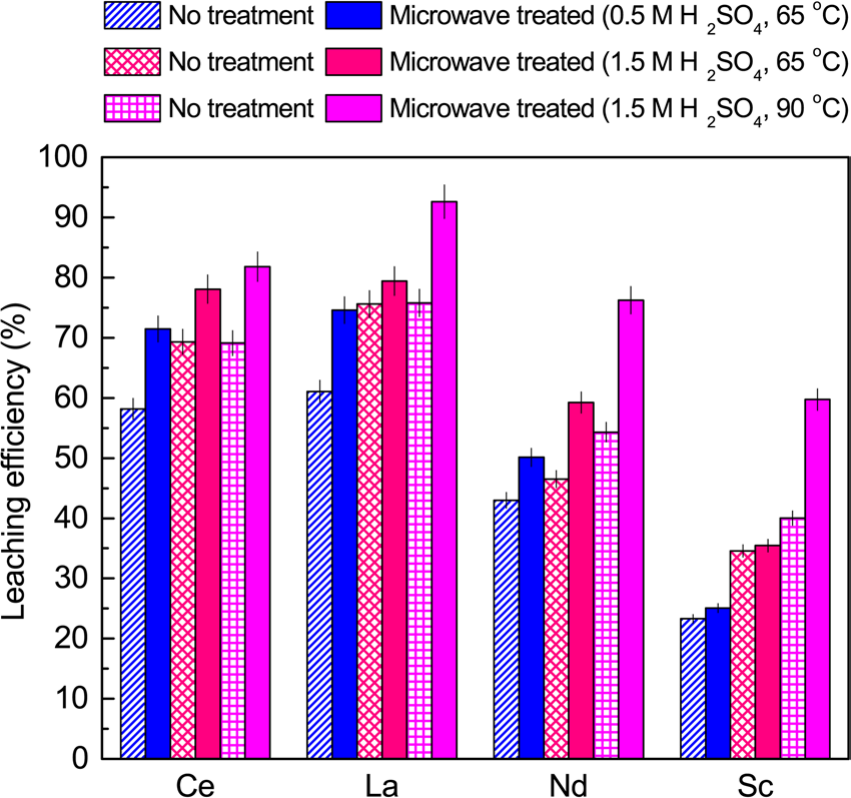
Effect of microwave pretreatment on the REEs leaching efficiency from bauxite residue (residence time = 30 min). Error bars represent the standard error of the mean for four replicates.

Here, we examined the effect of microwaving bauxite residue prior to leaching in acid. Theoretically, the dielectric heating of water molecules present in the sample by microwave radiation would vaporize them, causing the formation of breaks and pores in these particles as the vapor escapes. The leachant would then be able to penetrate and diffuse further into the bauxite residue particles, bringing more REEs into solution. In this study, we investigated the effect of microwave pretreatment in H_2_SO_4_ system under three sets of conditions; 0.5 M H_2_SO_4_ at 65 °C, 1.5 M H_2_SO_4_ at 65 °C and 90 °C. As shown in Fig. 4, in all cases, the leaching efficiency increased when microwave pretreatment was utilized. As can be seen from the figure, microwave pretreatment followed by leaching in 1.5 M H_2_SO_4_ at 90 °C resulted in highest leaching efficiency for the REEs of interest; 81.8% for Ce, 92.6% for La, 76.2% for Nd, and 59.7% for Sc.

**Figure 5:**
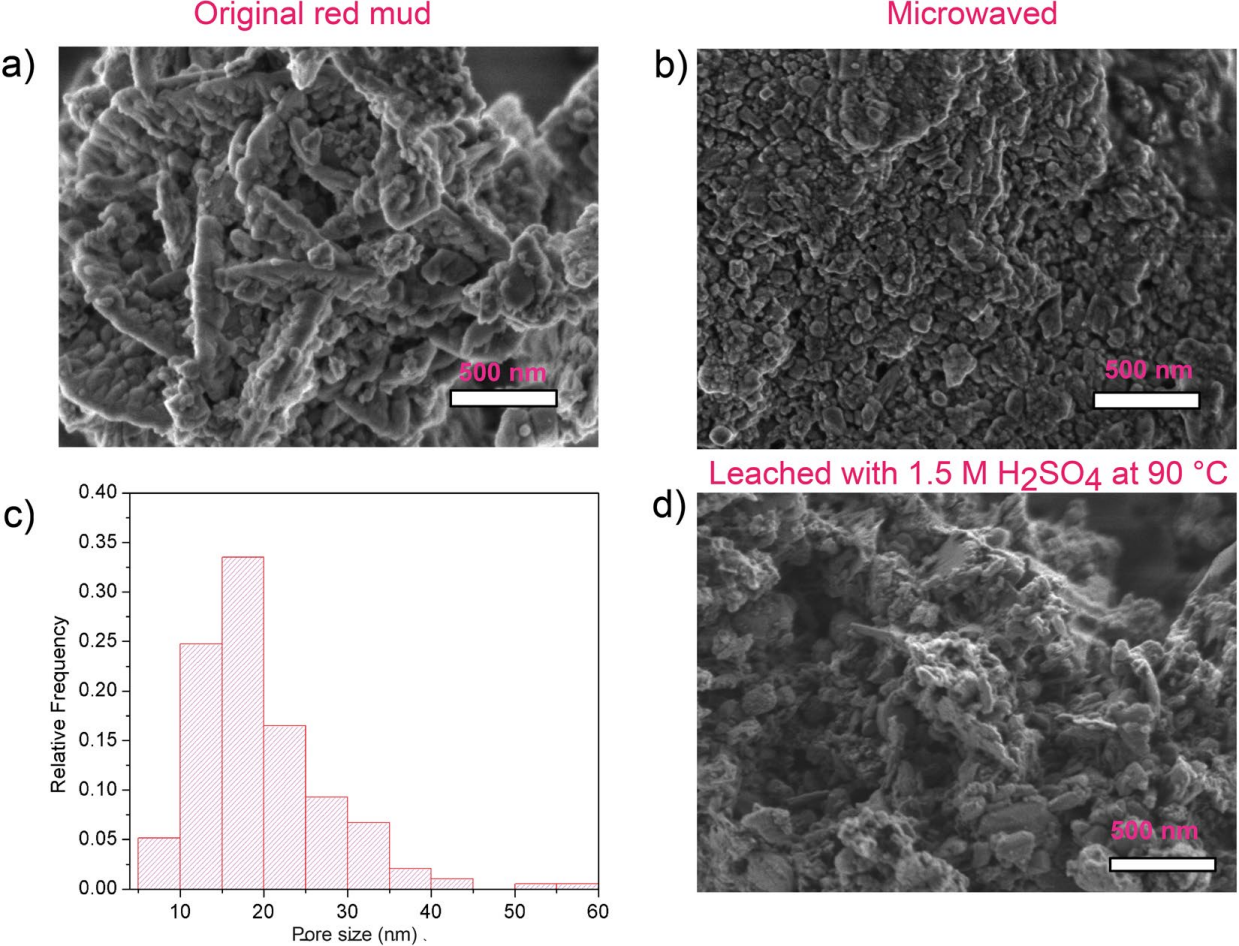
SEM images of (**a**) original bauxite residue; (**b**) microwave treated bauxite residue, nano-pores were developed; (**c**) histogram of pore size in microwave treated sample, and (**d**) bauxite residue leached in 1.5 M H_2_SO_4_ at 90 °C for 30 min.

To explore the changes in the surface structure, SEM was also performed on the bauxite residue after microwave pretreatment and leaching in 1.5 M H_2_SO_4_ at 90 °C for 30 min. An image of the original bauxite residue is included in Fig. 5(a) as reference. After the microwave pretreatment, nano-sized pores developed between the sub-particles on the surface of the bauxite residue particle (Fig. 5(b)). Pore size after microwave treatment was measured from multiple SEM images. On the basis of the histogram of the measurements, the average particle size was determined to be 20 nm (Fig. 5(c)). After leaching, a large portion of the sub-particles on the surface of the bauxite residue were detached, and the particle size decreased from 3.62 μm to 2.51 μm, resulting in a surface with larger scale porosity, as shown in Fig. 5(d). The surface area of the particles before and after microwave treatment was measured using BET (Brunauer–Emmett–Teller) technique, and the results indicated an increase from 21.76 m^2^/g to 27.77 m^2^/g, respectively. These observations suggest that the leaching agent did infiltrate into the bauxite residue particles through the pores developed from the microwave treatment and the increased in surface area also contributed to enhanced dissolution of REEs.

### Kinetics investigations and exploring mechanism

**Figure 6:**
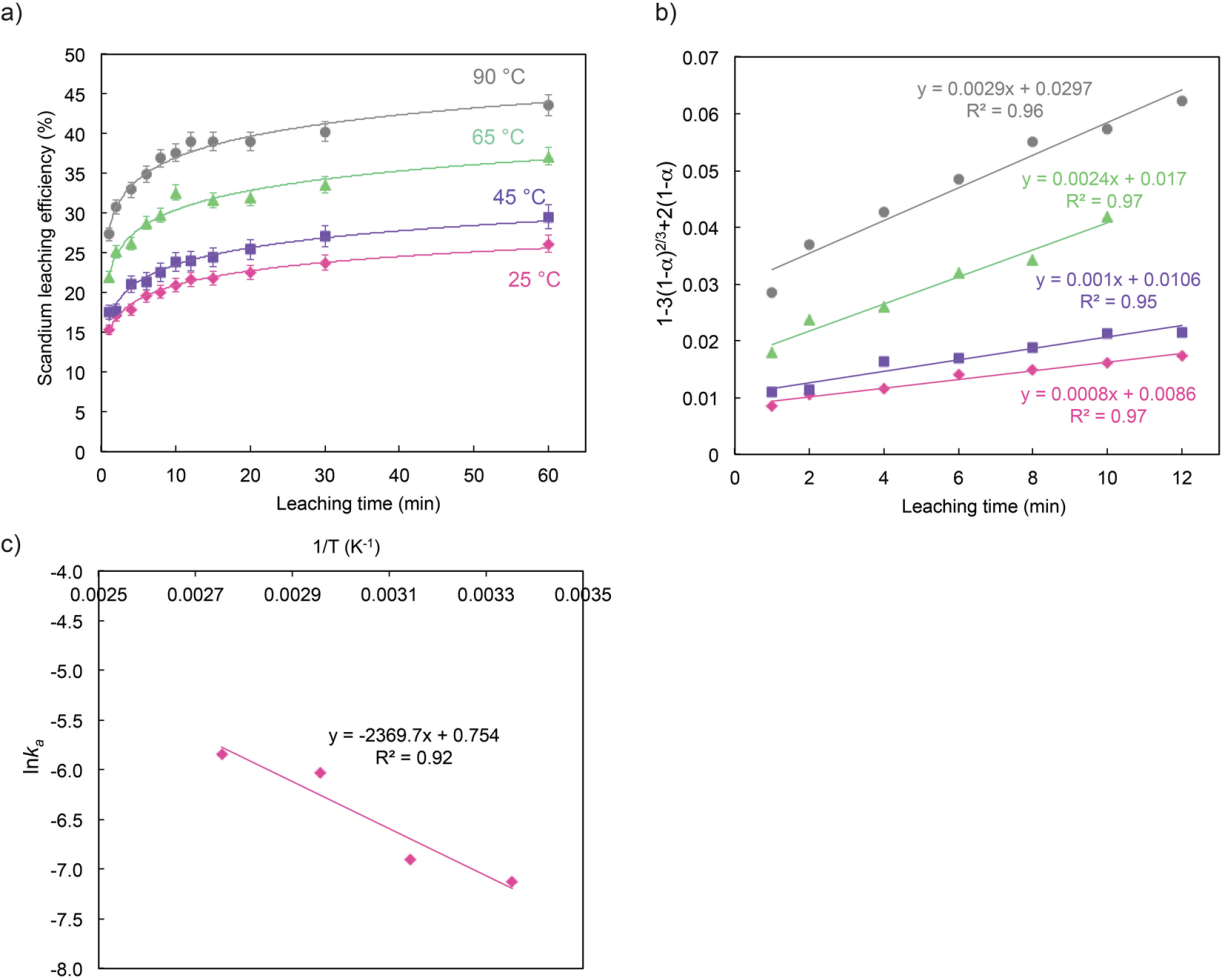
(**a**) Leaching efficiency of scandium (Sc) as a function of time at various temperatures using 1.5 M H_2_SO_4_ as the leaching agent. (**b**) Plots of $[1-3{(1-\alpha )}^{\frac{2}{3}}+2(1-\alpha )]$ versus time at various leaching temperatures. (**c**) Arrhenius equation plot to determine the apparent activation energy (*E*_*a*_) of the leaching reaction. Error bars represent the standard error of the mean for four replicates.

To uncover the mechanism of REEs leaching process from bauxite residue in the H_2_SO_4_ system (without microwave pretreatment), liquid samples were taken over time up to 60 min at all four temperatures. For modelling purposes, we put the emphasis on scandium. As can be seen in Fig. 6(a), at the initial stage of leaching for all four temperatures, the leaching rate increases rapidly with increasing leaching time, and then the increase slows down and reaches a plateau. It can also be seen that increasing the leaching temperature improves the leaching process. To investigate the scandium leaching kinetics, we adopted the shrinking core model, which plays an important role in the fluid-solid systems^22,23^. The equations for this model are as follows:

1 $$1-{(1-\alpha )}^{\frac{1}{3}}={k}_{a}t$$

2 $$1-3{(1-\alpha )}^{\frac{2}{3}}+2(1-\alpha )={k}_{a}t$$

where *α* is the leaching efficiency of the element at time *t* (min) and *k*_*a*_ is the apparent reaction rate (min^−1^). Eq. (1) describes the leaching process of the solid particles with spherical geometry when rate controlling step is the chemical reaction, while Eq. (2) represents the case in which the diffusion of the reagent through the boundary layer is the rate controlling step. The plots of $[1-3{(1-\alpha )}^{\frac{2}{3}}+2(1-\alpha )]$ versus leaching time using the data of Fig. 6(a) resulted straight lines (presented in Fig. 6(b)) with the adjusted coefficient of determination (R^2^) more than 0.95, suggesting that the leaching of bauxite residue is controlled by the diffusion step. On the basis of the apparent reaction rate constant (*k*_*a*_) from Fig. 6(b), the Arrhenius equation (Eq. (3)) was employed to determine the apparent activation energy of the reaction.

3 $$k=AExp(-\frac{{E}_{a}}{RT})$$

where *A* is the frequency factor, *E*_*a*_ is the apparent activation energy (J.mol^−1^), R is the universal gas constant (8.314 J. mol^−1^.K^−1^) and T (K) is absolute temperature. Plotting ln*k* as a function of 1/T resulted a straight line with R^2^ equals to 0.92 (Fig. 6(c)). Using the slope of the line, the apparent activation energy for leaching of scandium from bauxite residue using 1.5 M H_2_SO_4_ was calculated to be 19.70 kJ/mol. According to the literature, the apparent activation energy of the diffusion-controlled leaching is ~20 kJ/mol; whereas it is greater than 40 kJ/mol for a chemical reaction-controlled leaching^9^. Thus, the value of the apparent activation energy further supported that the rate-controlling step of the leaching of bauxite residue is the diffusion of the leaching agent through the boundary layer. These results are consistent with the results of a previous study that showed a diffusion-controlled reaction for REEs leaching from phosphogypsum (a REE-containing residue produced by the fertilizer industries)^24^.

It should be mention that, an acceptable fit (R^2^ of 0.85) of $1-{(1-\alpha )}^{\frac{1}{3}}$ versus time could also be achieved based on the data presented in Fig. 6(a); however, the apparent activated energy using the Arrhenius equation was calculated at 11.9 kJ/mol ( < 20 kJ/mol); thus, the chemical reaction was not the rate-controlling step.

**Figure 7:**
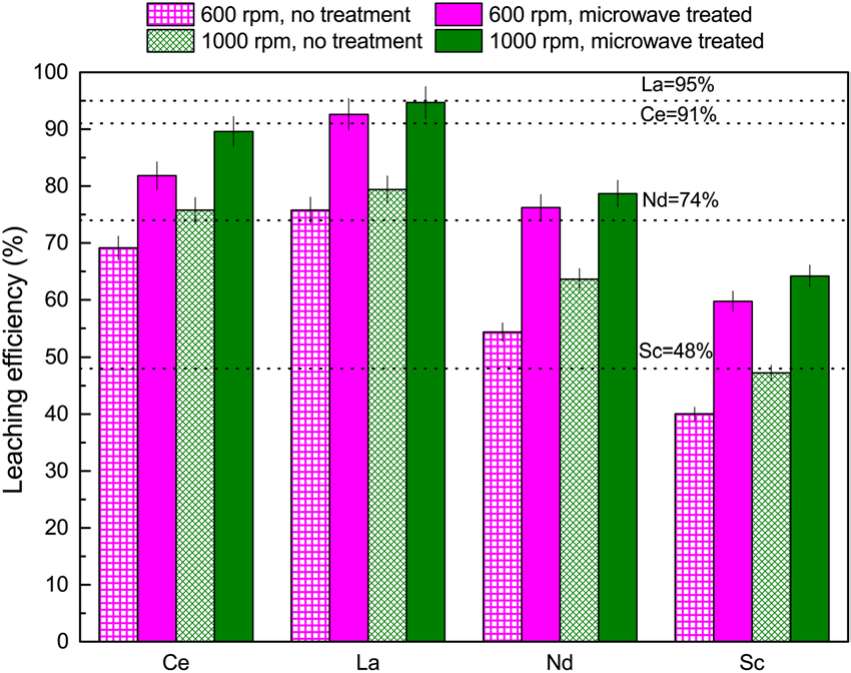
The effect agitation on the leaching efficiency of REE from bauxite residue in 1.5 M H_2_SO_4_ at 90 °C (with and without microwave pretreatment) within 30 min residence time. The dashed lines represent the maximum leaching efficiency that could be achieved using 3 M HNO_3_ at 90 °C without microwave pretreatment. Error bars represent the standard error of the mean for four replicates.

In this study, we also studied the effect of agitation rate and improved mass transfer on the leaching efficiency by increasing the agitation of the reactor from 600 rpm to 1000 rpm (leachant: 1.5 M H_2_SO_4_, 90°C, with and without microwave pretreatment). As depicted in Fig. 7, the leaching efficiency increased with the stronger agitation in the reactor. The dashed lines in Fig. 7 presents the maximum leaching efficiency for each REE (Ce = 91%, La = 95%, Nd = 74%, Sc = 48%) that was obtained when 3 M HNO_3_ was used at 90 °C (without microwave pretreatment). We have demonstrated that with the combination of microwave pretreatment and increased slurry agitation in the reactor, the leaching efficiency of REEs in H_2_SO_4_ were similar to or even outperformed leaching in HNO_3_.

## Conclusions

We have demonstrated a simple, ambient pressure acid leaching process to recover REEs from Canadian bauxite residue. Three different leaching agents were investigated and H_2_SO_4_ was chosen to be the most suitable based on leaching efficiency, cost, and practical process considerations. Based on the kinetic model developed, the leaching process of REEs from the bauxite residue was controlled by the diffusion of leaching agent through the boundary layer. By exposing the bauxite residue to a microwave treatment prior leaching, nano-sized pores developed on the surface. The change in surface structure allowed the leachant to penetrate deeply into the bauxite residue, resulting in enhanced leaching efficiency of REEs, i.e. from 40.0% to 64.2% for Sc and from 54.3% to 78.7% for Nd. The results from this study establish the feasibility of recovering technologically important REEs from widely available bauxite residue generated from industrial alumina production.

## Methods

### Chemicals and materials

Bauxite residue samples were obtained from Rio Tinto, located in Jonquière, Québec, Canada. The sample was dried overnight in an oven at 50 °C. After the drying process, samples were analyzed with thermogravimetric analysis (TGA), and the water content was always below 5%. Hydrochloric acid (ACS Reagent grade, 36.5–38.0% assay), nitric acid (ACS Reagent grade, 68.0–70.0% assay), and sulfuric acid (Solution 3.0 M BDH®) were purchased from VWR and were used for preparing acid solutions for leaching process. Deionized water (0.055 *μ*S, Millipore) was used to make the solutions.

### Experimental design

Leaching experiments were conducted inside a 1 L glass reaction vessel and heating was provided through a heating mantel. Temperature was controlled within ±1 °C of the set point. The reactor slurry was kept suspended by a shaft stirrer (600 and 1000 rpm). Samples were withdrawn through a dip rubber tube using a syringe and filtrations were performed using 0.45 μm nylon syringe filters from VWR. They were diluted with 5% HNO_3_ and stored in sealed plastic test tubes at room temperature. These samples were analyzed with ICP-OES (Perkin Elmer Optima 8000) to determine the concentration of REEs, Al, Ti and Fe in the leached solution. Leaching experiments were initially conducted for 2.5 h, and after analysis it was found that the REE leaching reaches its maximum at 30 min and plateaus beyond this point; thus 30 min was selected as the optimum residence time. To calculate the leaching efficiency, we used each REE concentration in the leach solution and the mass of the solid used in the experiment to calculate the mass of extracted REE per unit mass of solid (ppm, i.e., mg/kg) and divided that value by the REE concentration in the bauxite residue (presented in Fig. 2a). Reproducibility tests (four independent experiments) showed that the experimentally measured data are accurate to within ±5%.

### Microwave activation process

Microwave activation was performed using a multi-power microwave oven (Panasonic, NN-ST775S). A known amount of bauxite residue was placed inside an alumina crucible. Bauxite residue samples were treated at 1000 W for 10 min. Microwave oven was purged continuously with 0.5 L/min of nitrogen gas. The temperature of the treated samples was measured using a K-type thermocouple (Omega). After microwaving, solid samples were collected and characterized by SEM. The microwave treated samples were then used as the feed for the leaching experiments.

### Characterization of the test specimens

#### Aqua Regia digestion and ICP-OES characterization

To determine the concentration of REEs in the bauxite residue sample, aqua regia digestion at 220 °C was performed, using an Ethos EZ Microwave Digestion System, followed by ICP-OES. Solution samples taken at each sampling time were also analyzed by ICP-OES. Four independent experiments were conducted to determine the average values of REE contents.

#### Morphological, compositional, and crystal structure and surface area BET analysis

Morphological characterizations of the samples were obtained with SEM (Hitachi SU8230) and STEM/EDS (Hitachi HF-3300). The mineralogical analysis of the sample was investigated with XRD (Philips PW1830). Surface area BET analysis was performed utilizing a Quantachrome autosorb gas sorption analyzer (model: Autosorb iQ-MP). All samples were outgased at 200 °C for 6 hours to remove any water and all analysis was later performed at 77 K (liquid nitrogen).
